# Models for Treating Post-traumatic Headache

**DOI:** 10.1007/s11916-021-00970-3

**Published:** 2021-06-14

**Authors:** Joshua Kamins

**Affiliations:** 1 Goldberg Migraine Program, Department of Neurology, David Geffen School of Medicine at University of California Los Angeles, Los Angeles, CA USA; 2 Steve Tisch BrainSPORT Program, Department of Neurosurgery, David Geffen School of Medicine at University of California Los Angeles, Los Angeles, CA USA

**Keywords:** Post-traumatic headache, Concussion, Traumatic brain injury, Headache, Persistent post-concussion symptoms

## Abstract

**Purpose of Review:**

To discuss the treatment of post-traumatic headache (PTH) and how to choose pharmacotherapy based upon known pathophysiology.

**Recent Findings:**

Preclinical models of traumatic brain injury are finally revealing some of the mechanisms of PTH, including the significant role that inflammatory neuropeptides like calcitonin gene-related peptide (CGRP) play in the initiation and persistence of symptoms.

**Summary:**

To effectively treat post-traumatic headache (PTH), one needs to understand the pathophysiology behind the initiation and persistence of symptoms. Recent animal models are starting to elucidate these mechanisms, but effective treatment will also likely rely on the identification of patients who are most at risk for persistent PTH. Trials of early, targeted therapy for at-risk patients will be needed to validate these hypotheses. Additionally, high powered clinical trials are lacking in the field of persistent PTH for medications that are known to be effective in primary headache disorders. Effective treatment for persistent PTH also requires understanding how headache interacts with the complex nature of persistent post-concussion symptoms, as this disease often necessitates a multi-disciplinary approach. Regardless, with the knowledge gained by new PTH models cited in this paper, and an increasing availability of novel headache medications, more effective treatment models are on the horizon.

## Introduction

In addition to being the most common acute symptom after traumatic brain injury (TBI), headaches are the most persistent and disabling symptom after mild TBI. In a 2006 review, Lew et al. reported 18 to 33% of TBI sufferers went on to experience post-traumatic headache (PTH) at 1 year. In a prospective cohort of concussion patients admitted to the hospital, 58% (109/189) were reported to suffer from headaches 1 year post injury, with migraine-like headaches being the most common headache type reported [1]. We are also starting to learn that not all PTH are the same. Specifically, PTH with a migraine phenotype signifies a worse outcome [2]. And yet, there is mounting evidence that the acute concussion syndrome and much of the known pathophysiology resolves within 30 days [3]. So, we must ask, why are post-traumatic headaches so refractory to treatment, and what can we do about them?

In order to have success in alleviating PTH, we must understand when and how to treat it. To accomplish this, we should be thinking about what treatment models we should employ, both evidence-based and novel, in acute and persistent PTH. We should consider the role of early treatment in order to prevent persistent PTH, and for whom this applies. The clinician should always keep in mind how PTH interacts with other symptoms of persistent post-concussion symptoms (PPCS), including significant emotional distress, physical deconditioning, and autonomic symptoms. Although the answers to these dilemmas are not always clear, there are clues in the pathophysiology of concussion and PTH, and the author believes that we are at an inflection point with exciting and new treatment models on the horizon.

## Acute PTH Treatment Models

There has historically been hesitation in the acute concussion field to treat headache early due to concern over the detrimental effects of masking symptoms and pushing patients back to activity too early. There is additional concern utilizing medication: if approximately 80% of patients recover spontaneously by 4 weeks, why prescribe medication with possible side effects and unclear efficacy? What we now understand from investigations into physical activity is that after an appropriate amount of rest for approximately 24-48 hours, early sub-threshold activity, both physical and cognitive, is beneficial for recovery [4••]. Therefore, one could hypothesize that early treatment of headache symptoms would facilitate earlier return to activity, decrease avoidant behavior, lower likelihood of pain catastrophizing [5] and emotional distress, and provide an overall smoother recovery. This is a significant shift from traditional concussion management that requires simply hoping that symptoms do not persist beyond a normal time frame of 2 to 4 weeks.

The only pharmacologic trials that have been successfully completed in the acute window utilized parenteral therapies on patients presenting to the emergency department with severe headache. Friedman et al. found that after intravenous metoclopramide and diphenhydramine, twenty out of twenty-one subjects with acute PTH reported improvement of their headaches to no more than “mild,” but one-third of subjects had headache relapse after discharge, and one-fourth still reported persistent headaches 1 week later [6]. Chan and colleagues retrospectively analyzed a cohort of 254 patients, between 8 and 21 years old, with mild TBI and PTH less than 2 weeks since injury. After receiving one or more intravenous medications (ketorolac, metoclopramide, prochlorperazine, chlorpromazine, and/or ondansetron), most (86%) of their patients achieved treatment success, with 52% reporting complete resolution of headache prior to discharge [7]. These two studies suggest some role for medications traditionally used in the acute treatment of migraine such as anti-inflammatories and phenothiazines.

Although the evidence for early treatment is limited, the author suggests considering early treatment of at-risk individuals given the potential delayed benefit of headache prevention medications. Identifying at-risk individuals remains a major challenge. The predicting and preventing postconcussive problems in pediatrics (5P) study was established to design a clinical risk prediction score for PPCS in children, though not PTH specifically [8]. Significant predictive factors included being adolescent or female, prior concussion with > 1-week recovery, diagnosis of migraine history by a physician, acute symptoms of headache, fatigue, phonophobia, or answering questions slowly, and a score of ≥4 on the balance error scoring system (BESS) in the ED. In a recent study by the Four Corners Youth Consortium, PTH with a migraine phenotype strongly predicted persistent headache at 3- and 6-months post injury, compared to PTH with a non-migraine phenotype. Although female gender has long been considered a risk factor for prolonged recovery after concussion, this cohort demonstrated that the discrepancy in persistent PTH by gender was explained by a higher likelihood of migraine phenotype in girls [9].

In order to understand targeted treatment, we should target known pathophysiology. Acute symptoms of concussion, including headache, sensory sensitivities, nausea, dizziness, brain fog, and mood disturbance, are likely related to the metabolic cascade [10] and its sequela that represent the pathophysiology of concussion—though it is unclear to what extent headache symptoms are related to each aspect of pathophysiology. Immediately after a sufficient acceleration-deceleration injury, neurons undergo a substantial influx of sodium and calcium and efflux of potassium via mechanoporation. The brain attempts to correct the disturbance in homeostasis via ATP-fueled pumps and channels leading to early hyperglycolysis. Concurrently, there is altered cerebral hemodynamics and mitochondrial dysfunction. Drugs and supplements that may ameliorate this metabolic crisis, like creatine monohydrate or ketones, represent an untapped and intriguing area of research for acute PTH.

In addition to this significant ionic flux, there is widespread release of glutamate, leading to a cortical spreading depression [11, 12]. The glutamatergic surge may activate the trigeminovascular system in a similar manner to a typical migraine headache. Drugs that target the glutamate system are of particularly interest in acute PTH. Topiramate, an inhibitor of the kainate subtype of glutamate receptors, is a commonly used medication in migraine prevention, although its side effect profile limits its utility in this context. Memantine, an activity dependent inhibitor of the NMDA receptor subtype of glutamate receptors, has some evidence for the prevention of migraine [13]. Additionally, given potential cognitive benefits and low side effect profile, it may be an ideal drug in this patient population. Magnesium is also an activity-dependent blocker of NMDA receptors, and multiple studies have demonstrated magnesium may play a role in migraine [14].

Subsequently, there is an inflammatory component with microglial activation, upregulation of cytokines, and a release of pain-signaling molecules including inflammatory neuropeptides with role in pain and headache like calcitonin-gene-related-peptide (CGRP), pituitary adenylate-cyclase-activating polypeptide (PACAP), and substance P [15]. The literature is strongly pointing toward CGRP, a neuropeptide known to play a substantial role in the generation of migraine [16], as a primary generator of acute post-traumatic headaches. A number of animal models have demonstrated elevation of CGRP acutely after TBI, corresponding to symptoms of pain and light sensitivity. Additionally, treatment that targets this CGRP elevation appears to alleviate these symptoms [17].

One of the most meaningful recent acute PTH studies in animals demonstrated that administration of an anti-CGRP monoclonal antibody at 2 h and 7 days after mild TBI blocked injury-induced allodynia and partially prevented bright light stress-induced allodynia [18••]. Administration of the antibodies on day 10, after the resolution of the allodynia, did not prevent bright light stress-induced cutaneous allodynia. This suggests that early CGRP sequestration may be much more effective than delayed treatment once central sensitization has been established and pain becomes independent of CGRP.

CGRP elevation can be targeted by triptans, gepants (small-molecule CGRP antagonists), CGRP monoclonal antibodies, and even NSAIDs [19] or corticosteroids [20]. As noted above, acute reduction in CGRP has been shown in animal models to decrease the likelihood of the development of delayed sensitization. Current insurance requirements make targeted CGRP intervention with antibodies and gepants difficult in acute PTH. However, for patients at a high risk of developing persistent symptoms based on clinical risk scores, and perhaps, given the work of Kamins et al. [9], those who have PTH with a migraine phenotype, urgent efficacy studies are needed. In their absence, the author has found that employing corticosteroids, NSAIDs, or long lasting triptans like frovatriptan for a 5-7 day course may be effective to significantly diminish symptoms and shorten the acute symptom course after concussion.

## Persistent PTH Treatment Models

The mechanism of persistent PTH is even less clear than acute PTH. The acute concussion physiology noted above generally resolves within 4 weeks [21]. And yet, persistent PTH occurs in a significant number of individuals after mild TBI. Most studies report that migraine is the predominant phenotype of persistent PTH, followed by tension-type headache. Is persistent PTH as some suggest, simply migraine induced by trauma, representing an interaction of biological risk factors and trauma in susceptible individuals? Or, is it a completely separate headache disorder with overlapping symptomatology? Important studies are working to tackle this dilemma, including preclinical models [22] as well as clinical investigations utilizing imaging studies [23] and detailed phenotypic analysis of PTH [24].

Preclinical models of persistent PTH pathophysiology are relatively sparse, as compared to acute TBI models. However, a recent preclinical model of persistent symptoms demonstrated that while CGRP is highly implicated in the acute window, it also may play a role in increasing sensitivity to migraine triggers in the persistent phase. Fourteen days after a rat model of closed head injury, when the initial cephalic hypersensitivity had resolved, cephalic sensitivity could be triggered again by low-dose glyceryl trinitrate, a known migraine trigger. This was then inhibited by sumatriptan or an anti-CGRP antibody. Persistent PTH may therefore represent a state of persistent sensitivity to headache triggers, mediated by CGRP [25]. However, as Navratilova and colleagues describe, once central sensitization has set in, there are certainly CGRP-independent physiologies as well. Similarly, while glutamate is highly implicated in the acute phase of TBI leading to cortical spreading depression, there may also be persistent changes to glutamate receptor composition [26].

Clinically, the recommendation remains to treat the headache based on the phenotype it most closely represents. This author has anecdotally observed that for many patients, concussion triggers an underlying risk for a primary headache disorder like migraine. These patients may have a family history of migraines but have never expressed it, or have a very mild and occasional headache which is significantly exacerbated by their injury. The mechanism of this is both physiological–related to neurotransmitter and neuropeptide changes—as well as due to lifestyle changes, including worsened sleep, decreased physical activity, and increased anxiety and depressive symptoms. In other patients with no premorbid risk factors, it may be a wholly unique headache syndrome, often with continuous pain and no sensory dysfunction. Finally, in a large subset of patients, there is a combination of central sensitization marked by daily or continuous pain with allodynia and photosensitivity, with superimposed paroxysmal migraine-like headaches. Clinical features that may differentiate migraine from PTH include an increased rate of autonomic dysfunction [27], higher levels of anxiety and depression, and in the author’s experience, more frequent continuous pain and allodynia.

The treatment of persistent PTH is just starting to accumulate evidence. Even in specialty concussion clinics, providers appear relatively reluctant to initiate headache specific treatment. We can see from Pearson et al. [28] how providers are currently treating PTH in youth, with many physicians waiting to initiate therapy for 8 or even 12 weeks after injury. Popular treatments include vitamins/supplements, amitriptyline, and topiramate. This is likely partially related to a lack of evidence for more advanced therapies.

Vitamins and supplements can be particularly appealing in a disease that particularly afflicts youth and adolescents. While melatonin initially showed promise based upon a retrospective chart review of patients with PPCS [29], a subsequent randomized, placebo-controlled study could not find a significant effect of melatonin when compared with placebo in an intention-to-treat analysis [30].

With regards to prescription preventive medications, Cushman et al. reviewed the effect of gabapentin and tricyclic antidepressants for persistent PTH in a retrospective analysis of 277 patients with headaches after concussion from their sports-medicine clinic. Both treated and untreated patients recovered over time. Subgroup analysis demonstrated that headache significantly decreased in the visit following initiation of gabapentin, but no continuous decrease in headache was observed thereafter. The TCA group did have a significant improvement in symptoms from the first follow-up appointment to the last visit. However, the treatment group had higher baseline symptom scores than the untreated group, making the results difficult to interpret [31].

Known to be particularly effective in chronic migraine, botulinum toxins are an attractive treatment strategy to target PTH with a high headache frequency. Zirovich examined the efficacy of botulinum toxin A in a pilot trial of persistent PTH patients and found that it led to a significant reduction in headache days per week as compared to the placebo group [32]. This reinforces a retrospective analysis in military veterans with PTH demonstrating benefit of on a botulinum toxin despite the majority of their patients having continuous pain [33].

As discussed previously, CGRP appears to have a role in acute PTH and potentially persistent PTH, and CGRP targeted treatment feels especially promising when a migraine phenotype is present. In a small case series of 5 women with persistent PTH, patients reported a mean reduction of headache intensity of 51.1% with no adverse events [34]. In a non-randomized, 12-week, open-label study of 100 individuals with persistent PTH, with a mean of 15.7 days per month of moderate-severe headache, there was a mean reduction in moderate-severe headache days of 2.8 per month, with 28% subjects reporting > 50% reduction in their moderate-severe headaches [35].

There is little to no evidence for the efficacy of triptans, NSAIDs, or gepants for abortive treatment of persistent PTH. However, one aspect of PTH treatment that the author has found useful is employing corticosteroids, non-steroidal anti-inflammatories, or occipital nerve blocks to break the patient’s headache cycle at the onset of implementing preventive medications. This helps decrease central and peripheral sensitization that may be occurring and give the preventive medication a higher likelihood of being effective. Gepants and long-acting triptans may also have some utility. Dr. Conidi recommends a similar strategy in which he utilizes an 18-day steroid taper and often adds a concurrent 9-day course of triptans or gepants [36].

In addition to the medications discussed thus far (TCAs, gabapentin, botulinum toxin, CGRP MaBs, NSAIDs, steroids, and triptans), other acute and preventive headache medications that have not been studied may have significant value given our experience with them in other headache syndromes, and good anecdotal response in clinic patients. These could include beta blockers, candesartan, serotonin-norepinephrine reuptake inhibitors (SNRIs), topiramate, zonisamide, pregabalin, and memantine.

## Special Considerations

Patients with post-traumatic headaches present certain treatment challenges compared to those with primary headache disorders.

1. Given the abundance of sports-related concussions, it is vital to account for the patient’s sport of choice when initiating treatment. Medications that limit heart rate, notably beta-blockers and calcium-channel blockers, will significantly decrease cardiac output and limit exercise tolerance. The author strongly recommends avoiding these medications in athletes. Additionally, providers should be aware of banned substances by sports organizations like World Anti-Doping Agency (WADA) which, for example, bans beta-blockers during competition in a number of sports.
2. Patients with persistent post-concussion symptoms frequently report cognitive symptoms. Therefore, providers should be aware of medications that may trigger or exacerbate cognitive symptoms, and in certain patients, avoid these medications altogether. While this primarily includes topiramate, medications with significant cholinergic side effects like amitriptyline, and other sedating medications, like the gabapentinoids, should also be used with caution.
3. As previously discussed, patients with PTH may have comorbid autonomic dysfunction. Providers should take this into account when prescribing medications that can worsen orthostatic tachycardia like tricyclic antidepressants and medications that can exacerbate postural hypotension like beta blockers.
4. High rates of anxiety and mood disorders in patients with PPCS and PTH can be disabling and disruptive to care. Medications that can dually treat depression and headache, such as duloxetine, can be particularly useful in these patients, while others that can exacerbate depression like flunarizine and topiramate may need to be avoided altogether. Careful titration is also encouraged when prescribing compounds with stimulating effects, such as SNRIs or memantine, in those suffering from uncontrolled anxiety.
5. Concurrent medical treatment of headaches and peripheral pain sources, including cervicogenic etiologies, may benefit from noradrenergic medications (e.g., duloxetine, nortriptyline) or gabapentinoids.
6. Finally, always be on the lookout for secondary headaches that can also be trauma-induced, including intracranial hypotension secondary to cerebrospinal fluid leak [37] and TBI with intracranial pathology.

## Not Just a Headache: Cervicalgia

Most mechanisms of traumatic brain injury also exert force upon the cervical spine, and attention should be paid to peripheral pain sources, including cervical facet joints, ligaments, muscles, tendons, intravertebral disks, and spinal/peripheral nerves. While headache is primarily transmitted through the trigeminal system, it is clear that the upper cervical nerve roots (C1-3) and the rostral cervical spinal cord may play critically important roles in the generation of headache [38]. Peripheral nerve blocks like greater (GON) and lesser (LON) occipital nerve injections with a local anesthetic, with or without steroids, may help reduce nociceptive afferent feedback to the trigeminal nucleus caudalis. Studies in PTH have been promising, although none have been randomized, placebo-controlled trials. One case series in a pediatric population of persistent PTH found that out of 15 patients who received them, 9 reported long-term response to occipital nerve blocks, with associated improvement in quality of life and decreased post-concussion symptom scores [39]. Given the rapid benefit of nerve blocks, occipital nerve injections are an attractive and safe method for both acute and persistent PTH management. Aside from occipital nerve blocks, targeted treatment of other peripheral pain sources may be beneficial, including trigger point and tendon injections, facet joint injections, or medial branch blocks [40]. Physical therapy for any cervical injury may also be vital to ameliorate muscular and postural imbalances and restore normal body movement patterns.

## Multi-disciplinary Approach

In the author’s experience, one surefire way to fail in treatment of PTH is to provide medication without additional support. Post-traumatic headaches are multi-dimensional and often part of the larger persistent post-concussion symptom picture [41]. A multi-disciplinary team, capable of treating this multi-faceted disease, will provide a significantly higher chance of success.

Neuropsychologists are essential team-members in the evaluation of PTH patients’ cognitive and emotional state, and if needed, clinical psychologists should be available to provide psychotherapy and coping strategies. Symptoms of anxiety and depression are tragically common after traumatic brain injury [42]. This is important to recognize for effective treatment of PTH for a number of reasons. Primarily, anxiety and depression may contribute to worsened prognosis in headache disorders [43], and are associated with poor sleep quality, deficient nutrition, and decreased exercise. Additionally, other emotional distress symptoms, including pain catastrophizing, lead to a higher likelihood of persistent post-traumatic headache [44]. Finally, some symptoms of anxiety disorders, including PTSD, may mimic symptoms of PTH, including sensory dysfunction [45•].

Specific to the field of PTH, patients’ fear over inducing headache from physical activity (kinesiophobia) or cognitive activity (cogniphobia) are associated with significantly increased disability [46]—in the author’s experience one of the largest barriers to recovery. This is often reinforced by well-intentioned but ill-informed providers who have previously instructed patients to fully cognitively and physically rest until their symptoms resolve. Subthreshold exercise has been found to be of significant benefit in recovering not just from acute concussion but also persistent post-concussion symptoms [47]. At our institution, we have paired cognitive behavioral therapy and subthreshold aerobic exercise with great success, enabling patients to become minimally symptomatic in a controlled setting, with a psychologist and occupational therapist present to help them utilize mind-body techniques to allow for increased exercise workload [48]. There is also evidence that aerobic exercise diminishes the burden of migraine [49] with proposed mechanisms including upregulation of brain-derived neurotrophic factor (BDNF), improved neurovascular regulation, and modulation of endogenous pain fighting systems.

Sensitization to light, sound, and other sensory input may also respond well to exposure therapy as an option for those patients who have become significantly avoidant of these stimuli and have actually exacerbated their sensitivity due to this avoidant behavior [50]. Occupational therapists can also play increasing roles in concussion recovery to help patients reengage with activities that they have been avoiding, including exercise, school, and work [51].

Often, it does not matter how skillfully executed your medication regimen is, if the patient is not sleeping, unable to play the sport they love, is avoidant of their teammates and friends, and overall are not able to enjoy life, their headaches are frequently refractory to treatment. Whereas when pharmacotherapy is combined with education and cognitive restructuring, aerobic reconditioning, management of sleep disturbance, and when appropriate, psychotherapy, then medication can appropriately target physiologic causes of post-traumatic headaches with renewed efficacy.

## Future Studies Needed

The future has to be big, because the present is too small. Clinical trials will be on the horizon for new medications.

More investigator-initiated trials for CGRP antibodies will likely be coming, and hopefully soon we see high-powered, randomized, placebo-controlled trials as well. The gepants, given their role as acute CGRP receptor antagonists, also represent an intriguing method for CGRP blockade in the acute post-traumatic timepoint. Because we are currently using them as acute migraine medications, and now with data demonstrating efficacy in migraine prevention [52], investigation of the gepants for the treatment of acute post-traumatic headache and prevention of persistent PTH will be an exciting next step.

New drugs, yet to come to market, make physiologic sense as well. Similar to CGRP, PACAP is a neuropeptide that is elevated both in migraine and after TBI [53, 54]. Monoclonal antibodies to the PAC1 receptor are in early phase clinical trials as a potential therapy for migraine. As with the CGRP antibodies, there is a strong rationale for considering antibodies to the PAC1 receptor or to PACAP itself as potential therapies for post-traumatic headache.

Neuromodulation devices have been undergoing significant advancement in the primary headache world, including, but not limited to, repetitive nerve stimulators, noninvasive vagus nerve stimulators, and supra-orbital nerve stimulators. Given the involvement in PTH of the trigeminal system, similar to migraine, the supra-orbital nerve stimulator merits particular investigation. Additionally, with the increased rate of systemic autonomic symptoms in patients with PPCS and PTH, vagus nerve stimulation is a logical modality to explore.

However, as discussed, the highest need may not be finding new treatments, but finding the right patients and the right timing. If we can predict whose headaches will persist beyond 1 month, and who would benefit from early intervention, we likely have a number of effective medications ready to deploy. Constructing longitudinal cohorts that document post-traumatic headache phenotype, while pairing them with therapeutic relevant biomarkers, may be the key to identifying which of our patients merit early treatment and targeted pharmacotherapy.

## Conclusion: Recommended Treatment Algorithm

**Fig. 1:**
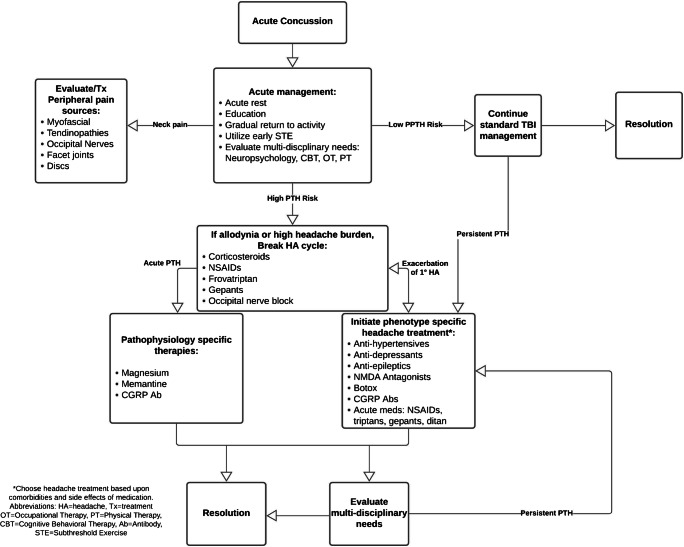
Hypothetical treatment algorithm

A proposed treatment algorithm for PTH is summarized in Fig. 1. Acutely, one should apply appropriate post-TBI care models by educating patients about their expected course and utilizing hyper-acute rest with an early but gradual return to physical and cognitive activity. Acute PTH should be treated aggressively in patients who are experiencing significant headache-related disability, and extra attention should be paid to patients with factors that put them at significant risk of persistent PTH: high acute symptom scores, personal history or strong family history of migraine, and PTH with a migraine phenotype. Early pharmaceutical and procedural treatment options may include NSAIDs, triptans, steroids, gepants, and peripheral nerve blocks. If evidence supports it in the near future, utilization of pathology-specific headache preventive treatments may be warranted early, including CGRP antibodies and NMDA antagonists. Providers should evaluate for and appropriately utilize methods to reduce central and peripheral sensitization such as NSAIDs, steroids, nerve blocks, and antidepressants with noradrenergic activity. Subsequently, acute and preventive therapies should be selected based on the headache phenotype and medication side effect profiles. Ideal preventive medication options may include tricyclic antidepressants, SNRIs, memantine, candesartan, botulinum toxin, and CGRP antibodies—avoiding medications that present higher side effect profiles in this patient population. Lastly, utilize pharmacologic and non-pharmacologic strategies within a multi-disciplinary team to treat comorbid symptoms that contribute to disability or interfere with recovery, including aerobic deconditioning, autonomic dysfunction, anxiety, depression, and/or sleep disturbance.
